# Rural hospital incident command leaders’ perceptions of disaster preparedness

**DOI:** 10.1186/s12873-025-01201-3

**Published:** 2025-03-18

**Authors:** Jason P. Murphy, Clara Bergström, Lina Gyllencruetz

**Affiliations:** 1 Department of Diagnostics and Intervention, Umeå University, Umeå, 90187 Sweden; 2 Institution for Health Sciences, Swedish Red Cross University, Huddinge, Sweden; 3 Department of Nursing, Umeå University, Umeå, Sweden

**Keywords:** Hospital incident command, Disaster medicine, Disaster preparedness, Decision-making, Major incident, Rural preparedness

## Abstract

**Background:**

Recent trends indicate that the frequency of major incidents (MIs) is increasing. Healthcare systems are vital actors in societies’ responses to MIs. Well-prepared healthcare systems may mitigate the effects of MIs. Disaster preparedness is based on region-specific risk and vulnerability analyses (RVAs). Hospital incident command groups (HICGs) are commonly formed per hospital’s contingency plan MI to aid in disaster response. Acquiring situational awareness and decision-making in the face of uncertainty are known challenges for HICGs during MIs. However, the remoteness of rural hospitals presents unique challenges.

**Aim:**

The aim of this study was to explore HICG leaders’ perceptions of disaster preparedness in rural hospitals.

**Methods:**

A qualitative study with semi-structured, focus group, and individual interviews was used. The data were analyzed through inductive content analysis.

**Results:**

The analysis generated the main category, HICGs’ *confidence in handling major incidents* and four categories. These were *Uncertainty and level of recognition* (containing two subcategories); *Awareness of challenges and risks* (containing two subcategories); *Factors that facilitate preparedness*,* response*,* and leadership* (containing three subcategories); and *Prerequisites for decision-making* (containing three subcategories and four subcategories).

**Conclusions:**

HICG leaders generally perceived their hospital’s disaster preparedness as adequate. However, preparedness was found to be influenced by several factors. The findings revealed a complex interplay of factors influencing preparedness and response, particularly highlighting challenges related to geographical isolation and resource constraints. Effective preparedness requires a comprehensive understanding of local contexts, hospital capabilities, and risks, which directly impacts training, decision-making, and resource allocation. Addressing the identified vulnerabilities necessitates targeted interventions focused on situational awareness, decision-making, collaboration, and training.

**Clinical trial number:**

Not applicable.

**Supplementary Information:**

The online version contains supplementary material available at 10.1186/s12873-025-01201-3.

## Background

The frequency of major incidents (MIs) has increased in recent decades, with 2023 exceeding previous disaster-related mortality averages [1], placing increased pressure on national preparedness and response strategies [2]. An MI, defined as an event that overwhelms available resources and requires specific leadership to maintain normal levels of care [3, 4], necessitates robust disaster preparedness for effective response [5, 6].

Well-prepared hospitals may mitigate both the somatic and the psychological effects of MIs [3]. Several factors affect hospitals’ ability to respond to an MI, including adequately trained personnel, its surge capacity, and contingency plans [5, 7]. Surge capacity is defined as a hospital’s ability to increase its capacity to manage a sudden influx of patients [5]. Hospital contingency plans employ an all-hazards approach and are based on region-specific risk and vulnerability assessments (RVA) [8, 9]. The effective execution of these plans hinges on well-trained hospital incident command groups (HICGs) [10], which are typically activated in the early stages of an MI or when an imminent risk is perceived.

HICGs, comprising leaders such as incident commanders and key departmental representatives, provide strategic leadership crucial for effective disaster response [10, 11], including the ability to make appropriate decisions often based on scarce or unreliable information [3].

Real-life activation of contingency plans in Sweden is rare [6], rendering assessment of disaster preparedness and response a challenge. While previous research has assessed preparedness in urban settings through simulations, interviews and cross-sectional studies [6, 12–14] identifying factors such as training, organizational structure, and information access as critical to situational awareness and effective decision-making [12–14], few studies research have addressed the unique aspects of rural hospital preparedness.

A rural setting is defined as a population density below 150 inhabitants per km2 [15] Given Sweden’s geographic diversity, regions encompass varying degrees of urban and rural areas, resulting in disparities in healthcare availability [16]. This geographical variation implies that different regions may experience varying impacts in the event of an MI, influenced by resource availability and preexisting conditions, distances to healthcare facilities, the number and size of hospitals, and the density of physicians and nurses.

While previous research has focused on urban preparedness [12, 17, 18] or compared urban and rural settings [19–21], studies specifically addressing rural hospital response and preparedness in the current study setting are lacking [5]. This study, therefore, aimed to explore the perceptions of HICG members regarding disaster preparedness within two rural regions of northern Sweden.

## Methods

### Study design

This qualitative study was conducted through semi-structured focus group discussions and individual interviews.

### Setting and participants

The participants were selected from three emergency rural hospitals in two regions in northern Sweden. The hospitals in these regions serve as primary facilities for emergency care. Each region is characterized by populations composed of approximately 50% rural residents and low population density (2.7 and 5.1 residents per square kilometer) [22]. Each hospital is classified as an emergency care hospital, which is characterized as providing 24-hour emergency medical services and having emergency departments with at least two medical specialties, i.e., internal medicine and surgery [23]. The sizes of the three hospitals range from 80 to 280 hospital beds and 600-5,700 employees. The number of ICU beds ranges from approximately 7–25 [24], and each hospital has disaster preparedness and response plans.

**Fig. 1:**
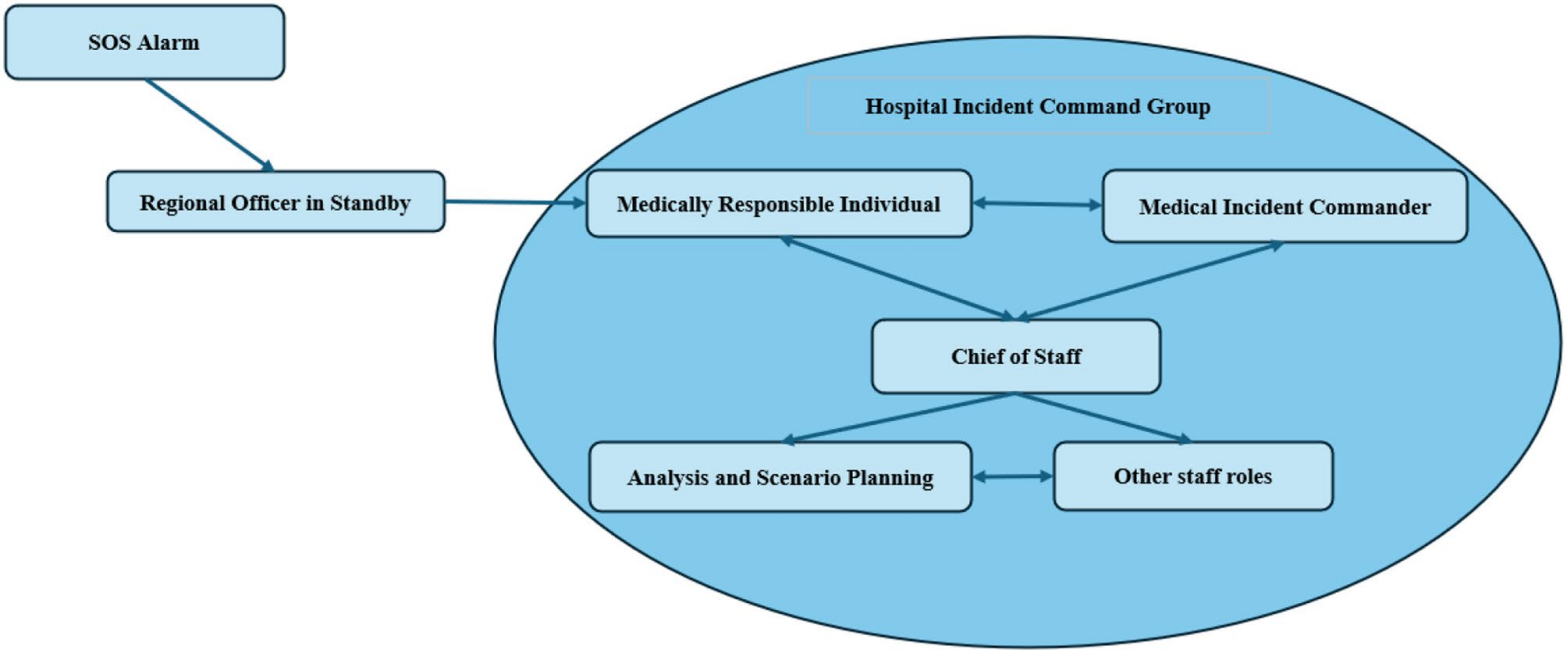
Relevant structure of the hospital incident command groups

A purposive sampling method was utilized to identify individuals from the hospitals’ HICGs. Hospital disaster preparedness coordinators for each hospital aided in the identification of participants according to the criteria set. The criterion set for inclusion was individuals in leadership or analytical roles within the HICG, with the authority to make strategic decisions. No criteria concerning formal training, education, or experience were included to ensure that all possible active members of HICGs were included. The relevant roles identified as relevant were chief of staff (CoS), medically responsible individual (MRI), medical incident commander (MIC), and analytical and scenario planning (ASP) (Fig. 1). Sample size was aided and assessed through information power in accordance with the five criteria as stipulated [25]: [1]. Study aim [2], sample specificity [3], quality of dialog [4], use of established theory, and [5] analysis strategy. The recruitment of participants was facilitated by local disaster preparedness coordinators. A total of 25 potential participants were contacted, with 15 from three different hospitals agreeing to participate (Table 1).

**Table 1:** Descriptions of participants

Participants (*n* = 15)					
Gender	Male	Female			
	9	6			
Current profession	Physician	Nurse	Other		
	6	5	4		
	Mean (years)	Range (years)			
Age	55	46–63			
Years in profession	26	2–42			
Years in HICG*	8	2–13			
Role in the HICG	MIC	MRI	CoS	ASP	Multiple roles
	3	3	5	1	3

### Data collection

The interviews were conducted with 15 members of the HICGs at three rural hospitals in two regions in northern Sweden. A semi-structured interview guide based on a previous study’s results [26] containing both open-ended and more specific questions was constructed by CB and JM, specifically for the study setting (Table 1). Data collection was conducted in two phases. The first phase consisted of three focus group discussions (FGD) consisting of a total of 12 participants and was conducted by CB and JM. The second phase consisted of individual interviews. These interviews were divided into replacement interviews for those unable to participate in the FGD and follow-up interviews. Due to the CoSs’ all-encompassing role of the HICG and knowledge, their specific insight was of particular interest; thus, follow-up interviews were conducted with incident CoSs by CB with JM consulting both prior to and after the interviews. In total, three focus groups and 12 individual interviews were held (Table 2). All the interviews were conducted through either Zoom^®^ (*n* = 11) or face-to-face (*n* = 4) and were recorded and transcribed verbatim by CB with JM, facilitating the focus group discussions by ensuring that the interview guide was adhered to and contributed questions as well as assisting in technical issues during the interviews (FGDs). This resulted in 8 h and 43 min of audio recordings. The transcripts and audio files were coded and anonymized.

**Table 2:** Interview type distribution

Interview type	Focus group *(n = 3)*	Follow-up *(n = 7)*	Replacement *(n = 5)*
Virtually	3	6	2
Face-to-face	0	1	3

### Data analysis

Inductive content analysis was utilized to analyze the data, due to the explorative nature of the study [27, 28]. To ensure trustworthiness, the three phases, as described previously [29], were employed: (I) preparation, (II) organization, and reporting (III). The preparation phase consisted of identifying the most appropriate participants based on the aim of the study as well as deciding to use inductive content analysis as the method for analysis. Upon completion of the interviews, the transcribed text was read through multiple times while listening to the recordings. In the organizational phase, initial open coding was conducted by CB and discussed between CB and JM back and forth until consensus was achieved. The codes were then grouped into categories, which were then grouped into higher-order categories. During the reporting phase, discussions concerning subcategories and categories were held until consensus was reached between CB, JM and LG. Investigator triangulation was used to minimize subjectivity and potential bias and to increase trustworthiness [28] (Table 3). During the analysis process, the research group carefully considered the criteria for information power. It was concluded that a sufficient level of information power was achieved due to the focused aim, specificity of the sample, analysis strategy, and quality of the dialog.

**Table 3:**
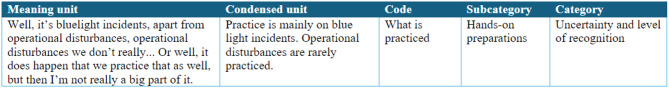
Example from the analysis process

## Results

**Fig. 2:**
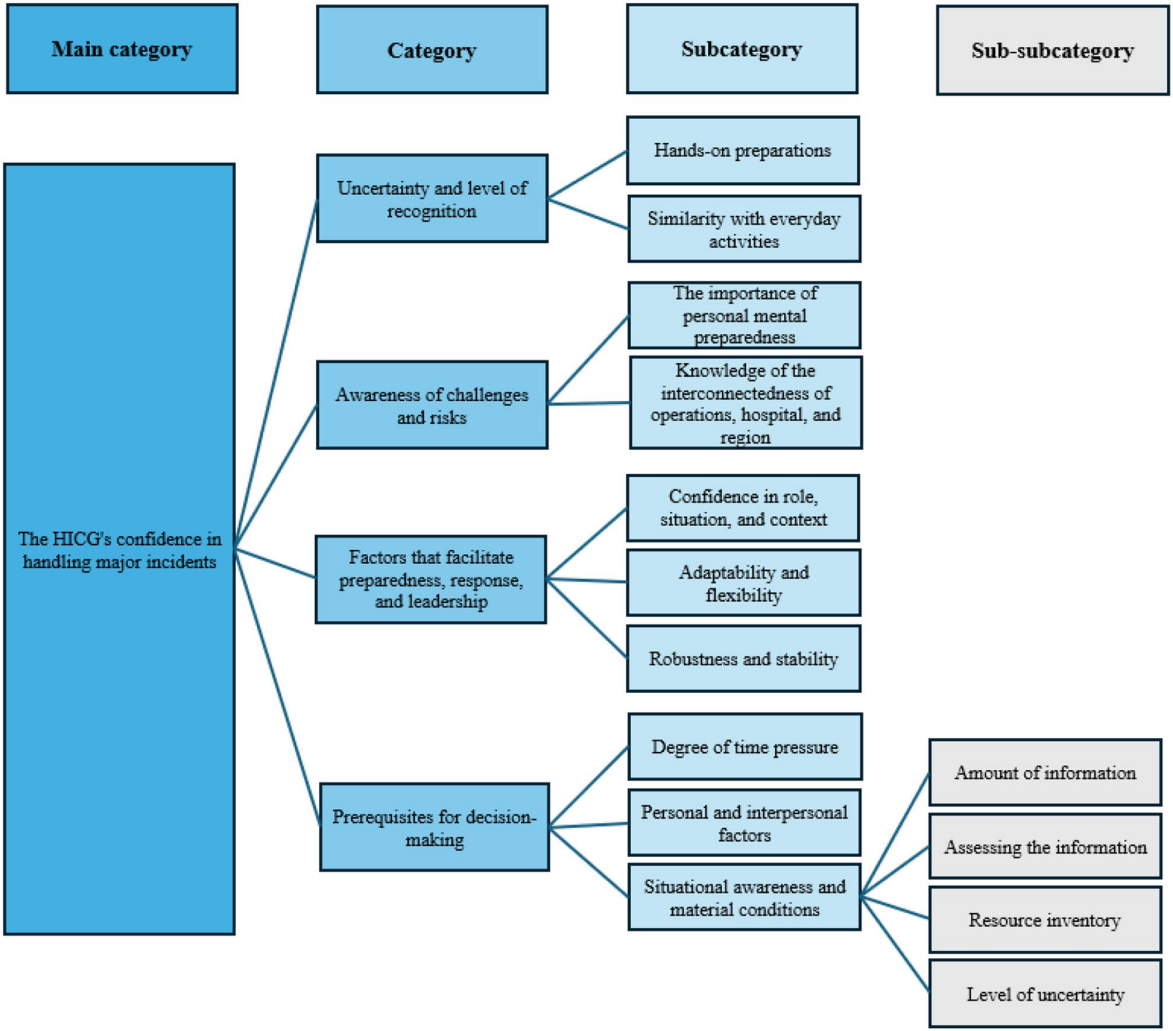
Results with main category, categories, subcategories, and sub-subcategories

The analysis of HICG members’ assessments of disaster preparedness yielded a primary category: HICGs’ confidence in handling major incidents. This was further categorized into four categories (illustrated in Fig. 2): *Uncertainty and level of recognition* (two subcategories); *Awareness of challenges and risks* (two subcategories); *Factors that facilitate preparedness*,* response*,* and leadership* (three subcategories); and *prerequisites for decision-making*.

### HICG’s confidence in handling major incidents

Analysis of the collected data revealed a main category: HICG confidence in managing major incidents. The participants generally perceived their hospitals’ and regions’ disaster preparedness as adequate. However, they also described disaster preparedness as complex, contingent upon their feelings of security and confidence in their ability to manage major incidents. This confidence was influenced by several factors, as detailed in the following categories.

### Uncertainty and level of recognition

The participants described that uncertainty, and the level of recognition are factors that affect their confidence in decision-making and preparedness. This category describes how hands-on preparations may mitigate uncertainty and how familiarity with situations facilitates management (Fig. 2).

#### Hands-on preparations

The participants indicated that hands-on preparations for major incident responses impacted their confidence and decision-making abilities. These preparations encompassed formal role-specific education, the quantity of training received, and prior real-life experience in managing similar incidents. The frequency of training was deemed particularly crucial, especially in contexts where training opportunities were limited.

*“You have got to practice to get good at this. And*,* every real incident is a learning experience.” (Participant 8) (Participant 8)*

A lack of relevant prior experience with n the HICG was associated with feelings of inadequate preparedness among participants. Conversely, prior hands-on experience with similar incidents aligned with better preparedness. Chemical accidents were highlighted as a particular area of concern, largely attributed to insufficient real-world experience and training. Participants without such experience either underestimated the threat or expressed uncertainty about their response capabilities. In contrast, participants with extensive experience in managing chemical incidents demonstrated greater confidence in their ability to handle similar major incidents, perceiving them as less threatening.

*“We’ve got [industries] which have a lot of chemicals*,* and we’ve had a couple of chemical simulations together*,* which was really good. And we’ve had some fire exercises as well*,* and simulations together with [the industries] […] and that’s been really valuable*,* since you get to learn together and learn who does what and communications channels.” (Participant 11)*

#### Similarity with everyday activities

Familiarity with incident types facilitates participants’ confidence in managing major incidents (MIs). The participants reported confidence in handling major incidents that are similar to incidents they encounter on a regular basis. For example, confidence levels were higher regarding traffic accidents, which are frequently encountered, than for chemical incidents, which are less common. Similarly, although more frequent than chemical incidents, extensive IT disruptions were viewed as challenging, as they fell outside the scope of typical hospital operations.

*“I’ll put it this way; I can feel safe when something happens similar to my everyday life.” (Participant 6)*

### Awareness of challenges and risks

This category describes the importance of being aware of the challenges and risks that HICG members might be forced to handle (Fig. 2). Being aware of potential risks was described as being a vital part of disaster preparedness.

#### The importance of personal mental preparedness

The importance of personal mental preparedness was emphasized by several participants. This included self-reflection on individual roles within the HICG, the hospital’s overall response strategy, and potential incident scenarios, as well as consideration of their individual contributions during major incidents. The participants attributed their feelings of security and reduced stress to adequate mental preparedness, whereas those lacking such preparedness reported increased anxiety about managing a major incident.

*“When I get the call*,* I usually take a few deep breaths on my way there because you do get an adrenaline rush when the call comes. And I work through the work method in my head and think ‘okay*,* what is it I have to do?’ and ‘what’s important here?’.” (Participant 12)*

#### Knowledge of the interconnectedness of operations, hospital, and region

The participants identified a comprehensive understanding of the local context—the “lay of the land”—as crucial for effective disaster preparedness. This included familiarity with hospital routines, infrastructure, capacity, and regional resources. Knowledge of the hospital’s role within the regional healthcare system provided valuable insight into its limitations. Furthermore, understanding the local geography, infrastructure, and industrial landscape facilitated a more thorough risk assessment, encompassing factors such as extended transportation times (road and rail) and potential hazards associated with local industries such as heavy machinery and risks associated with chemicals.

*“We’ve got quite a few risky operations nearby—various industrial plants*,* and we’ve even practiced scenarios involving the local mines. So*,* we definitely have a regional inventory process in place. And*,* it’s worth noting that there are two other hospitals—one in C and one in H—both approximately 130–150 kilometers away.” (Participant 11)*

### Factors that facilitate preparedness, response, and leadership

This category describes the factors identified that facilitate HICG preparedness, response and leadership. Participants described several factors positively impact the outcome of handling MIs, such as feeling confident as well as having the ability to adapt quickly. The following subcategories describe the different aspects that facilitate HICG performance (Fig. 2).

#### Confidence in role, situation, and context

The participants’ feelings of security and confidence within the HICG were influenced by several interacting factors. This sense of confidence was found to be context dependent, varying on the basis of individual roles, specific situations, or a combination thereof. Specifically, confidence in one’s assigned role stemmed from a clear understanding and level of comfort with the associated responsibilities.

*“Yes*,* I know the contingency plan. I’ve been a CoS both in my ordinary job and in the HICG for 10 years. We train frequently*,* with many simulations*,* and I’ve also been in the HICG in real events several times a year for 10 years’ time*,* so I would say I feel confident.” (Participant 8)*

The participants’ situational confidence reflected their belief in their own abilities, those of their colleagues, and the overall capabilities of the HICG. Crucially, the unique context of HICG operations—distinct from standard healthcare workflows—and confidence in established staff methodology were identified as central to this situational confidence, as these elements involve different roles and problem-solving approaches.

“I *would say I feel confident. We use this way of working*,* this form of staff management*,* we sometimes use it in our everyday as well.” (Participant 15)*

#### Adaptability and flexibility

Adaptability and flexibility—the capacity to respond effectively to changing circumstances, particularly during major incidents—were identified as key strengths. The participants noted that the smaller size of their hospitals, with fewer staff, fostered intimate knowledge of individual skills and capabilities, thereby facilitating the efficient assignment of personnel to appropriate roles. This close-knit environment also fostered a collaborative atmosphere, where staff were highly motivated to contribute, even with limited resources. A problem-solving and creative mentality was also reported to be prevalent in such settings.

*We usually solve most of it; we’re rather solution driven. […] We do have short routes of decision. It’s not very complicated. Then*,* we just have to make sure it works. That’s what we do in a small hospital.” (Participant 1)*

#### Robustness and stability

The availability of robust and stable resources is critical for effective major incident (MI) management. The participants emphasized that the capacity to handle MIs is directly linked to the robustness of both the hospital and the wider region; a well-resourced system is better equipped to manage major disruptions. Existing capacity constraints, even under normal operating conditions, significantly hamper the ability to cope with MIs. Robustness, as defined by participants, encompasses backup systems, adequate supplies, sufficient staffing for both hospitals and HICGs, and the ability to manage extended or protracted incidents. However, participants highlighted significant limitations in readily available resources (e.g., hospital beds, medical equipment), partly due to existing structural constraints within the healthcare system. Consequently, mass-casualty incidents were identified as posing the most significant challenges.

*“Stability concerning rolls and routines is key to handling a major incident. If we’re short-staffed*,* with lots of vacancies and not enough beds…well*,* it’s going to be tough to have any backup if things go south—whether it’s a war or a bus crash.” (Participant 4)*

### Prerequisites for decision-making

Numerous factors influence decision-making and response during major incidents. These include the specific circumstances of the event, the distance between the hospital and the incident site, individual and interpersonal dynamics within the HICG, and the challenges of assessing both information overload and a lack of reliable information. The participants highlighted a wide range of influences on decision-making, from highly personal factors to the overall level of situational understanding. This category details these factors and their impact on the HICG’s decision-making process during major incidents (Fig. 2).

#### Degree of time pressure

The urgency of emergency situations directly impacts the time pressure experienced by HICGs during decision-making. The participants described this urgency as a significant factor influencing decision-making processes. Time constraints often led to decisions made with incomplete information or higher levels of uncertainty. Furthermore, the distance between the hospital and the incident site was identified as a key determinant of time pressure; more distant incidents afforded more time for information gathering and deliberation.

*“I think it depends a bit on the distance to the incident site. Because if it’s far away*,* then it might be unnecessary to activate the state of disaster and call in everyone*.* […] But I think the time factor has great importance in this matter.” (Participant 11)*

#### Personal and interpersonal factors

Personal and interpersonal factors significantly influence HICG decision-making processes. This subcategory examines participants’ experiences, highlighting the impact of individual dispositions, personal perspectives, and group dynamics within the HICG. Mutual trust and effective collaboration were identified as essential for optimal HICG performance.

*“Even though we each have specific roles*,* it’s a lot easier to make decisions when you trust and know the people you’re working with.” (Participant 10)*

Communication challenges, both technical and interpersonal, emerged as significant obstacles. Inconsistencies in terminology across regions, hospitals, and municipalities hindered clear communication and coordination, potentially impairing the HICG’s response to major incidents.

*“I discovered that the municipalities use different terms for preparedness levels than we do at the hospital. Our lowest standby level is the same term they use for their highest one. […] It really frustrates me because it creates a huge risk that we won’t be able to communicate properly with each other.” (Participant 11)*

#### Situational awareness and material conditions

Reliable situational awareness is crucial for effective decision-making during major incidents. The participants described several factors influencing the attainment of situational awareness. A consensus emerged regarding the critical role of situational awareness in effective decision-making; its absence resulted in uncertainty and hampered decision-making processes. The following subsections detail four key factors affecting situational awareness (Fig. 2).

*“If we don’t have proper situational awareness*,* it’s difficult to make relevant goals [for the HICG’s work].” (Participant 7)*

##### Amount of information

Information from trusted sources is vital in attaining reliable situational awareness. In certain cases, information from official sources was scarce due to technical issues. This was seen as a barrier to gaining adequate situational awareness and was described by the HICGs as frustrating and difficult to handle. This often resulted in alternative information sources being utilized.

*“It’s always difficult with […] the initial phase*,* that’s what’s important but also difficult. Because there’s a lack of information*,* and it gets a bit disorientating before you get everyone on the right track*,* you know.” (Participant 8)*

Conversely, challenges also arose from information overload stemming from multiple sources, including onsite emergency services, the emergency room, media outlets, and social media. The preferred mitigation strategy involved prioritizing established communication channels to manage the influx of information. The participants emphasized the difficulty of navigating the simultaneous presence of information overload and a scarcity of reliable information, as discussed below.

*“I also believe it’s the extraordinary amount of information you get in the beginning*,* from different directions.” (Participant 6)*

##### Assessing the information

The participants found information assessment and processing to be challenging, particularly during periods of information overload from multiple sources. Effective organization and assessment of information were deemed crucial for achieving reliable situational awareness. While official communication channels (primarily radios) connecting onsite personnel were considered the most reliable source of information, social media, although viewed with scepticism, was acknowledged as a potentially relevant supplementary source. Discerning factual information from rumors also presents a significant challenge.

*“I wouldn’t look that much at social media. I would trust coworkers from my organization*,* preferably healthcare workers*,* since these are the people I work with every day.” (Participant 5)*

##### Resource inventory

Knowledge concerning available and potential resources is critical for effective HICG decision-making. Awareness of both onsite and regional resources directly influences resource allocation decisions. The lack of a readily available, comprehensive assessment of hospital capacity presented a significant obstacle to efficient resource and personnel allocation. The participants emphasized the importance of a robust resource inventory for effective MI response, given the centrality of resource allocation to the HICG’s role.

*“You should start taking stock of what the hospital has right now—it’s going to be needed soon enough. Start with the small stuff; it all adds up and will be useful*,* whether you’re dealing with ten patients or a hundred.” (Participant 5)*

##### Level of uncertainty

Uncertainty negatively affects situational awareness and often leads to reactive responses. The HICG’s understanding of an incident’s progression and the applicability of established routines are directly influenced by both situational awareness and effective decision-making. While familiarity with incident types enables proactive decision-making, drawing upon past experiences to inform calculated assumptions, a lack of familiarity necessitates greater reliance on reports from the incident site and often results in a more reactive response.

*“I guess it’s the level of knowledge*,* which can be very dependent on which type of incident happens. I mean*,* something you’ve never experienced before*,* it’s going to be very difficult [to handle].” (Participant 4)*

## Discussion

### Discussion of results

The factors influencing disaster preparedness and response in these rural hospitals are similar to those observed in larger facilities. However, this study reveals unique challenges faced by rural hospitals, particularly concerning proximity to incident sites and collaboration with other essential healthcare actors. These factors act as both facilitators of and barriers to effective disaster preparedness.

Rural hospitals may experience unique challenges due to geographical isolation and limited opportunities for collaborative responses [19]. A critical factor affecting surge capacity is resource availability [30], with the availability of resources to both rural and urban hospitals being inherently constrained. Therefore, effective response strategies (reverse triage, additional bed capacity, and cancellation of elective surgeries) are vital [19].

Previous research has noted that rural hospitals typically have fewer resources than their urban counterparts do, and their lower baseline occupancy rates further limit their surge capacity [21]. Although baseline resources per capita (surgical theaters and ICU units) may be greater in rural areas than in many urban areas, the potential for significant expansion beyond this baseline is often limited [31].

While rural hospitals rely on regional support systems for collaboration and resource allocation, the significant distances involved complicate rapid response [21]. The emphasis on self-reliance can be a challenge. Larger hospitals with higher baseline occupancy may be able to continue routine operations. In contrast, rural hospitals may be more dependent on transferring patients to increase capacity, which may cause adverse events [12]. Many participants expressed frustration with their limited influence on hospital structures, underscoring the need to address these systemic issues. This limited agency, coupled with feelings of isolation, represents a significant challenge for rural hospitals, particularly those often serving as the primary receiving points for patients from MIs.

Situational awareness also plays a vital role in decision-making. The ability of the HICG to make decisions impacts the management of the MI, which is consistent with findings from other studies [3, 13]. Uncertainty regarding an incident’s nature or extent adversely affects HICGs’ decision-making capabilities. The distance of rural hospitals from the incident site may facilitate situational awareness and minimize uncertainty. However, while a greater distance from the incident site may aid in attaining situational awareness, decision-making appears to be closely linked with HICGs’ knowledge. The level of knowledge and the amount of experience influence individuals’ confidence and decision-making perceptions. The greater the confidence felt by HICG members, the more proficiently they appear to be at managing an MI. Additional factors impacting decision-making (teamwork, stress) align with previous research [23]. Furthermore, the time aspect may allow other vital actions needed to increase hospital surge capacity, such as resource inventory, allocation of resources, initiating redistribution of patients, and establishing reliable communication with official sources.

While proximity may be a factor affecting hospital preparedness, mental preparedness was also identified as an important factor. The activation of the hospital incident command group in the study setting is rare [6]. This may explain why participants who frequently encounter disaster preparedness in their profession expressed a higher level of mental preparedness, thus enhancing their understanding and anticipation of potential risks. Furthermore, less experienced participants demonstrated less awareness and insight, likely due to their limited exposure and experience in handling such situations. A lack of insight and recognition of risks could negatively affect response, leading to uncertainty and reactive decision-making [12].

The more extensive a participant’s knowledge of their hospital and region was, along with their insight into specific risks and vulnerabilities, the more aware they seemed to be of potential challenges [6]. However, a lack of recognition of threats and risks remains a recognized challenge. Similar to findings from a previous study that indicated that hospitals downplayed the risks of chemical incidents, the results of this study suggest that there may be a lack of sufficient insight into the specific risks associated with mass incidents caused by major events, despite these being identified as regional risks [9, 31].

Previous studies suggest that time constraints and uncertainty often lead HICGs to enact the highest level of response, potentially with unintended consequences [19]. However, this study suggests that increased distance to the incident site might mitigate some of these negative effects, particularly by allowing for more time for decision-making and reducing information overload [4, 32]. This additional time may assist in reducing uncertainty through rational analysis and intuition-based decision-making [33], thereby potentially offsetting the surge capacity limitations often experienced by rural hospitals.

### Limitations/methodological considerations

This study included three hospitals in northern Sweden. Given the specificity and possible uniqueness of the study setting, the results may not be transferable to all rural hospitals. The number of participants was seen as a strength of this study. It has been suggested that information power is more relevant than the number of participants, minutes or saturation [25, 34]. Therefore, information power was used to determine if the sample size was sufficient. After discussion with all the authors, the inclusion criteria were set, and participants with unique knowledge and insight pertaining to the specific aim and area of research were recruited. Information power is reliant on the specificity of the aim, the specificity of the participants, the richness of dialog, and the analysis strategy. Upon assessing the criteria for information power, it was concluded that the information power for this study was sufficient [25].

The inclusion of group interviews as well as individual interviews is considered a strength. Individual interviews allow participants to voice opinions that they may not feel comfortable expressing in FGDs [35]. Complementing group interviews with follow-up interviews for some participants, specifically the chiefs of staff, was performed to address this and provide space for the individual’s perspective, as well as allow for discussions that were not covered in the group interviews. Replacement interviews were conducted with those who could not attend a group interview. This resulted in the inclusion of more participants (*n = 5*).

Prior experience and preunderstanding of the subject matter may aid in extracting data [36] but may be a source of investigator bias [37]. To minimize this and ensure trustworthiness, investigator triangulation was used when analyzing the data [38].

The transferability of this study is connected to information power. Information power was sufficient for this study and provides insights into the challenges faced by similarly situated rural hospitals; however, the results may not be transferable to all HICGs in rural northern Sweden.

## Conclusion

This qualitative study explored the perceptions of rural hospital HICG leaders regarding disaster preparedness. The findings highlight the complex interplay of factors influencing preparedness and response in rural settings, emphasizing the unique challenges presented by geographical isolation and resource limitations.

Effective disaster preparedness requires a comprehensive understanding of hospital capabilities, regional contexts, and local risks. This knowledge is fundamental to developing effective disaster readiness strategies, including training protocols and decision-making processes during major incidents. Disparities between perceived and actual risks represent critical vulnerabilities that must be addressed.

The study underscores the need for targeted interventions to enhance situational awareness, decision-making, and resource management in rural healthcare settings, emphasizing the importance of training, collaboration, and a robust understanding of local contexts. Further research should investigate these factors in broader contexts to inform effective strategies for enhancing disaster preparedness in rural and remote healthcare systems.

## Electronic supplementary material

Below is the link to the electronic supplementary material.

Supplementary Material 1

## Data Availability

No datasets were generated or analysed during the current study.

## Abbreviations

ASP: Analysis and scenario planning
CoS: Chief of staff
FGD: Focus group discussion
HICG: Hospital incident command group
MI: Major incident
MCI: Medical incident commander
MRI: Medically responsible individual
RVA: Risk and vulnerability assessment
