# Supplementary material for: Rural hospital incident command leaders’ perceptions of disaster preparedness
Source: BMC Emerg Med. 2025 Mar 18;25:45. doi: 10.1186/s12873-025-01201-3 (PMC11921675; doi:10.1186/s12873-025-01201-3)
Supplement: Supplementary file 1 — Supplementary Material 1 [file 12873_2025_1201_MOESM1_ESM.pdf]

Appendix 1. Interview guides

Interview guide – group interviews

What is your role in the HICG? (presentations)

How have you practiced? Types/format, which scenarios, how often?

What experiences do you have of major incidents?

What is a possible worst-case scenario of a major incident for your hospital or region?

What factors are there that affect how you get situational awareness?

What factors are there that affect decision-making?

What would it mean for your region and hospital if Sweden were to raise its national level of preparedness? What is your assessment of your ability to do that?

Possible scenarios posed if staggering discussions:

- New Year's at a popular skiing resort. Reports of fire and explosion in one of the main buildings. Malicious intent cannot yet be excluded. Lots of info on social media.
- Power outage due to bad weather in most of the municipality, including the hospital. Not clear when the power will return.
- Snowstorm. Traffic casualty with a truck carrying hazardous cargo. Probable leakage of cargo. Bystanders are starting to feel sick.

Interview guide – individual interviews

What official training have you had for your role in the HICG?

How familiar are you with the local contingency plan?

How safe do you feel in your role in the HICG?

What experience do you have with being in the HICG? When, what type of MI? What worked well and not so well?

What is your overall view of the disaster preparedness in your hospital and region?

What are the biggest challenges to the region's disaster preparedness?

Additional questions posed in replacement interviews:

- How have you practiced? What, how, how often?

- What factors are there that affect situational awareness?

- What factors are there that affect decision-making?
